# Exploring the association of two oxygenators in parallel or in series during respiratory support using extracorporeal membrane oxygenation

**DOI:** 10.5935/0103-507X.20220299-en

**Published:** 2022

**Authors:** Livia Maria Garcia Melro, Yuri de Albuquerque Pessoa dos Santos, Luis Carlos Maia Cardozo Júnior, Bruno Adler Maccagnan Pinheiro Besen, Rogério Zigaib, Daniel Neves Forte, Pedro Vitale Mendes, Marcelo Park

**Affiliations:** 1 Intensive Care Unit, Hospital Samaritano Paulista - São Paulo (SP), Brazil.; 2 Intensive Care Unit, Discipline of Clinical Emergencies, Hospital das Clínicas, Faculdade de Medicina, Universidade de São Paulo - São Paulo (SP), Brazil.

**Keywords:** Acute respiratory distress syndrome, Extracorporeal membrane oxygenation, Hypoxia, Hypercapnia, Decarboxylation, Oxygenators, Swine

## Abstract

**Objective:**

To characterize the pressures, resistances, oxygenation, and decarboxylation efficacy of two oxygenators associated in series or in parallel during venous-venous extracorporeal membrane oxygenation support.

**Methods:**

Using the results of a swine severe respiratory failure associated with multiple organ dysfunction venous-venous extracorporeal membrane oxygenation support model and mathematical modeling, we explored the effects on oxygenation, decarboxylation and circuit pressures of in-parallel and in-series associations of oxygenators.

**Results:**

Five animals with a median weight of 80kg were tested. Both configurations increased the oxygen partial pressure after the oxygenators. The return cannula oxygen content was also slightly higher, but the impact on systemic oxygenation was minimal using oxygenators with a high rated flow (~ 7L/minute). Both configurations significantly reduced the systemic carbon dioxide partial pressure. As the extracorporeal membrane oxygenation blood flow increased, the oxygenator resistance decreased initially with a further increase with higher blood flows but with a small clinical impact.

**Conclusion:**

Association of oxygenators in parallel or in series during venous-venous extracorporeal membrane oxygenation support provides a modest increase in carbon dioxide partial pressure removal with a slight improvement in oxygenation. The effect of oxygenator associations on extracorporeal circuit pressures is minimal.

## INTRODUCTION

The use of venous-venous extracorporeal membrane oxygenation (VV-ECMO) as rescue therapy for refractory hypoxemia in acute respiratory distress syndrome (ARDS) has increased worldwide.^(1-3)^ In VV-ECMO, the extracorporeal transmembrane oxygen transfer depends primarily on ECMO blood flow, while the carbon dioxide (CO_2_) clearance depends mainly on sweep gas flow.^(4,5)^ Arterial blood oxygenation depends on a more complex interaction among recirculation, ECMO blood flow, oxygenator function, patient cardiac output (CO), and pulmonary shunt.^(6)^

In clinical scenarios presenting with hyperdynamic status, standard VV-ECMO support might not be sufficient to correct hypoxemia and hypercapnia. Venous-venous extracorporeal membrane oxygenation refractory hypoxemia and/or hypercapnia rescue maneuvers include body temperature control, prone positioning, beta-blockade, neuromuscular blockade, and inhaled nitric oxide.^(7)^ In case of failure of these rescue maneuvers, the addition of a new oxygenator to the ECMO circuit or an additional circuit to the patient are possible strategies.^(8,9)^ However, in the current literature, there are only a few case reports about this issue, and there are no data about the gas exchange efficacy or blood pressure/flow consequences of in-series or in-parallel oxygenator associations during VV-ECMO support.^(10-16)^

Thus, this study aimed to characterize the pressures, resistances, oxygenation, and decarboxylation efficacy of two oxygenators associated in series or in parallel during VV-ECMO support.

## METHODS

This manuscript is part of a sequence of experiments conducted on porcine respiratory ECMO support, some of which were previously published elsewhere. This experiment was approved by the Institutional Animal Research Ethics Committee of the *Hospital Sírio-Libanês* in São Paulo, Brazil and was performed according to the National Institutes of Health Guidelines for the use of experimental animals.^(5)^

### Animal comfort and instrumentation

We studied five female domestic Agroceres pigs. Instrumentation and surgical preparation were performed as previously described.^(5,6,17)^ We assessed the comfort of the animals hourly or when necessary through the evaluation of the absence of unexplained tachycardia, unexplained hypertension, and any motor or vegetative reaction to a slight nociceptive stimulus applied to the animal’s snout.

### Animal procedures and data collection

At the end of instrumentation, the animals remained without further manipulations for a period of 60 minutes for stabilization. The extracorporeal circulation was then turned on with a sweep gas flow of zero and a blood flow of 1,500mL/minute. After 30 minutes, we collected blood pressure data from the venous line (P1), preoxygenator (P2) and postoxygenator (P3). Pressures were collected without sweep gas flow and with a range of blood flows from zero to 5,500mL/minute. The blood flow was raised in steps of 500mL/minute waiting one minute for stabilization and data collection in each step. After this first stage, lung injury and septic shock with multiple organ failure (MOF) were induced as described in a previous manuscript.^(5)^ Later, a second set of pressures was collected using the same methodology. Transmembrane pressure was defined as the preoxygenator pressure minus the postoxygenator pressure.

We used the collected data along with the animal clinical variables during both moments in the final analysis in conjunction with the mathematical modeling that will be described further. The extracorporeal oxygenation system used in the experiment was the Permanent Life Support System (Jostra - Quadrox D, Maquet Cardiopulmonary, Hirrlingen, Germany).

### Mathematical modeling and formulas

We have previously described a multicompartmental mathematical model.^(18)^ The background of oxygenation modeling was high-rated flow oxygenators. The rated flow of an oxygenator is defined by the amount of hypoxemic blood (oxygen saturation < 75%) that can be nearly fully saturated (95 - 100%) per minute.^(19)^ Therefore, in our primary analyses, we assumed that blood passage through the oxygenator results in 100% hemoglobin saturation by oxygen independent of blood flow, hemoglobin level (since it is in a normal range), or preoxygenator oxygen saturation. With this assumption, the in-series configuration would only result in an increment in oxygen dissolved in plasma, while the in-parallel configuration results in a decrease in blood flow to each oxygenator, and the flow shared by the oxygenators is inversely proportional to their resistances.

The decarboxylation rationale of in-series modeling was the effect of two consecutive passages of blood flow in the oxygenator. Importantly, the effect of a lower preoxygenator CO_2_ partial pressure on CO_2_ transfer was considered during passage through the second oxygenator.^(5)^ The gas flow was kept the same as the initial flow in each oxygenator, which is a real-life practice. The in-parallel modeling effect on CO_2_ transfer was based on single oxygenator CO_2_ exchange properties (a low effect of blood flow on CO_2_ transfer and a high effect of sweep gas flow on CO_2_ transfer). Therefore, the reduction in blood flow expected due to the parallel configuration would have a low impact on CO_2_ transfer. On the other hand, the presence of two oxygenators exposes the ECMO blood flow to fresh gas flow twice as often, once each oxygenator is habitually (in the bedside practice) kept with the same initial gas flow, resulting in a doubled sweep gas flow effect on CO_2_ transfer.

For all modeling, the sweep gas was considered as pure oxygen (fraction of inspired oxygen - FiO_2_ = 1)

The formulas used in the computations were as follows:

**Table:** 

Oxygenator resistance R (dynes x sec/cm^5^) = (preoxygenator pressure in mmHg - postoxygenator pressure in mmHg) ^*^ 80/ECMO blood flow in L/minute Resistance of in-series association (dynes x sec/cm^5^) = resistance of oxygenator 1 (dynes x sec/cm^5^) + resistance of oxygenator 2 (dynes x sec/cm^5^) (1/resistance of in-parallel association (dynes x sec/cm^5^)) = (1/resistance of oxygenator 1 (dynes x sec/cm^5^)) + (1/resistance of oxygenator 2 (dynes x sec/cm^5^))

### Data analyses and statistics

To explore the impact of oxygenator associations in series or in parallel, the following procedures were performed:

1. First, the resistances of oxygenators were calculated as a single oxygenator and later in both studied conditions (in series and in parallel). The association of the oxygenators was not actually tested. For each of the five animals, oxygenator resistance was calculated with eleven ECMO blood flows, both before and after the induction of MOF, giving a total of 110 estimates of oxygenator resistances. To assess the effect of in-parallel oxygenators, one estimate of resistance was randomly selected from this pool of 10 estimates (two per animal) for a given ECMO blood flow to use for one oxygenator and then replaced. Then, a second estimate of resistance was selected randomly from the 10 estimates (for the same blood flow), used for the second oxygenator, and then replaced. This was repeated 100 times, giving 100 pairs of resistances for each ECMO blood flow tested. The resistance behavior was then plotted according to the ECMO blood flow.
2. The resulting preoxygenator pressures were also calculated and plotted according to the type of association and the ECMO blood flow. For this calculation, the postoxygenator-associated blood pressure was considered the same as a single oxygenator. This assumption was adopted to keep the same arterial cannula pressure gradient (a condition necessary for a stable cannula blood flow).
3. The resulting transmembrane pressure was also calculated and plotted as single and associated oxygenators.
4. Simulating the effect on oxygenation, the postoxygenator oxygen partial pressure (PaO_2_) was plotted against the ECMO blood flow, as well as the total oxygen content in this postoxygenator position. The final effect on oxygenation (the resulting main variable) was measured through the resulting arterial oxygen saturation. All simulations were performed as a marginal model, holding constant the following variables: cardiac output, oxygen consumption (VO_2_), hemoglobin level, arterial CO_2_ partial pressure, pulmonary shunt fraction, and ventilator settings. To clarify the impact of VO_2_ on arterial oxygen saturation, two high values were used, one of VO_2_= 200mL/minute and another of VO_2_ = 300mL/minute. These variables are described in the figure legends.
5. Decarboxylation was simulated as described above, and the resultant arterial CO_2_ partial pressure was plotted against the ECMO blood flow.

We present the animal data as the median [interquartile range 25% - 75%]. The data comparisons before and after MOF induction were compared using Wilcoxon’s test. To improve the visibility of the trends of a given variable across the ECMO blood flows, we used Tukey´s median running smoothing technique to plot the central tendency measure.^(20)^ The significance level used was p < 0.05. R free source software version 4.0.5 was used for the mathematical and statistical calculations and graphs.^(21)^

## RESULTS

The median weight of the animals was 80 [79 - 81] kg. The general characteristics in both clinical conditions (baseline and MOF) of the animals are shown in table 1S (Supplementary material), in which we can observe that despite the lower hemoglobin level and higher central venous pressure in the MOF condition, the extracorporeal system pressures were similar between the baseline and after MOF establishment.

**Figure 1:**
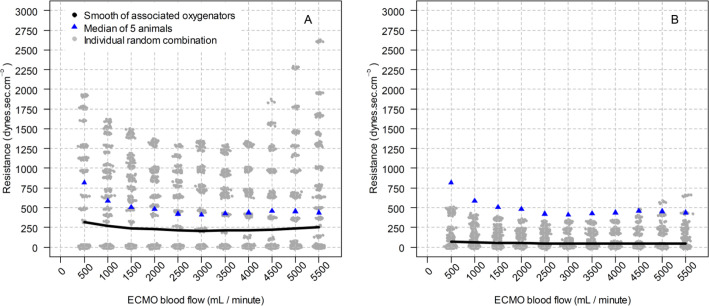
Median resistance for single oxygenators of five animals and resistances for 100 random associations of two oxygenators. (A) the in-series association and (B) the parallel association.

**Figure 2:**
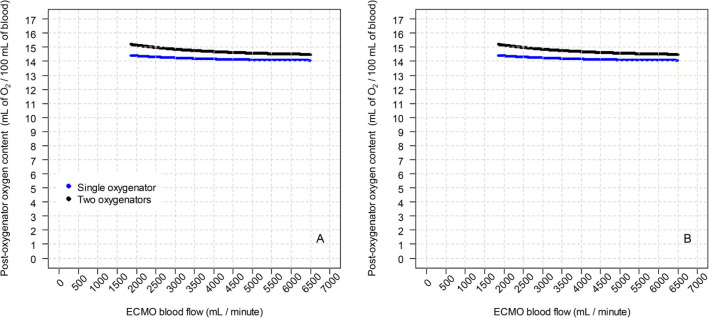
Post-oxygenator oxygen content using a single oxygenator and two parallel oxygenators. (A) The in-series configuration and (B) the parallel configuration.

**Figure 3:**
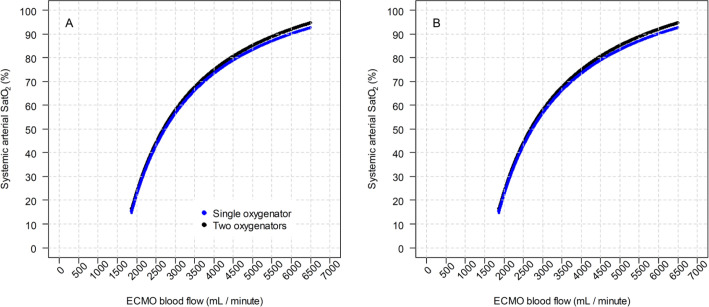
Systemic arterial oxygen saturation using a single oxygenator and two oxygenators. (A) The in-series configuration and (B) the parallel configuration.

**Figure 4:**
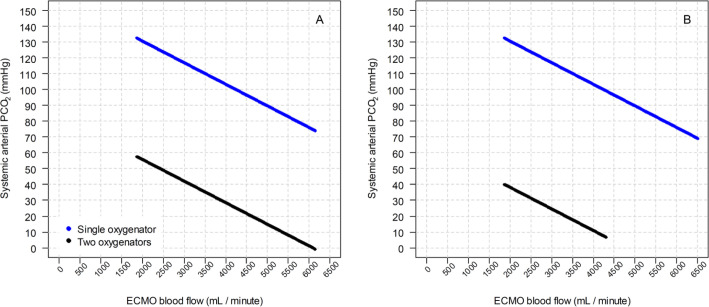
Systemic arterial carbon dioxide partial pressure using a single oxygenator and two oxygenators. (A) The in-series configuration and (B) parallel configuration.

To explore the mechanical features of the associations, figure 1 shows the calculated resistance of single oxygenators, in-series association (Panel A) and in-parallel association (Panel B) of two oxygenators, and a flat ‘U’ behavior with progressive high ECMO blood flows was observed. Figures 1S and 2S (Supplementary material) show the single oxygenator preoxygenator pressures and the resulting in-series and in-parallel preoxygenator pressures, respectively, according to the ECMO blood flow. Figures 3S and 4S (Supplementary material) show the single oxygenator transmembrane pressures and the resulting in-series and in-parallel transmembrane pressures, respectively, according to the ECMO blood flow.

For each extracorporeal membrane oxygenation blood flow point, there were 100 random combinations. ECMO - extracorporeal membrane oxygenation.

The oxygenation modeling is shown in steps. Figure 5S (Supplementary material) shows the effect of two oxygenators in series in the postoxygenator oxygen partial pressure (PaO_2_) dissolved in the plasma, according to the ECMO blood flow. Figure 6S (Supplementary material) shows the effect of two in-parallel oxygenators in the postoxygenator PaO_2_. Figure 2 shows the effect of the two tested configurations on the postoxygenator blood oxygen content, according to the ECMO blood flow. Figure 3 shows the main result of the modeling, that is, the two different configurations impact the systemic arterial oxygen saturation. Figures 7S and 8S (Supplementary material) show the systemic arterial oxygen saturation according to the ECMO blood flow with a higher VO_2_ (300mL/minute). The clinical controlled variables are shown in the figure legends.

Clinical status of modeling: oxygen consumption of 200mL/minute; hemoglobin level of 10g/dL; cardiac output of 10L/minute; partial pressure of carbon dioxide of 40mmHg; ventilator fraction of inspired oxygen of 30%; pulmonary shunt fraction of 100%; sweep gas flow of 4L/minute; sweep gas fraction of inspired oxygen of 100%. ECMO - extracorporeal membrane oxygenation. O2 - oxygen; ECMO - extracorporeal membrane oxygenation.

Clinical status of modeling: oxygen consumption of 200mL/minute; hemoglobin level of 10g/dL; cardiac output of 10L/minute; partial pressure of carbon dioxide of 40mmHg; ventilator fraction of inspired oxygen of 30%; pulmonary shunt fraction of 100%; sweep gas flow of 4L/minute; sweep gas fraction of inspired oxygen of 100%. ECMO - extracorporeal membrane oxygenation. SatO_2_ - arterial oxygen saturation; ECMO - extracorporeal membrane oxygenation.

The CO_2_ modeling is shown in figure 4, in which the systemic arterial PaCO_2_ is plotted against the ECMO blood flow in both configurations. The clinical controlled variables are shown in the figure legends.

Clinical status of modeling: oxygen consumption of 280mL/minute; hemoglobin level of 10g/dL; cardiac output of 10L/minute; pH of 7.40; sweep flow of 3.5L /minute; core temperature of 37ºC; pulmonary shunt fraction of 100%; sweep gas fraction of inspired oxygen of 100%. PCO_2_ - carbon dioxide partial pressure; ECMO - extracorporeal membrane oxygenation.

## DISCUSSION

In this study, we aimed to estimate the impact of two different configurations of VV-ECMO oxygenators on arterial oxygenation, CO_2_ removal, resistance, and blood pressure. In summary, we found that both oxygenator associations resulted in small changes in circuit blood pressures and systemic oxygenation and modest changes in decarboxylation efficacy.

### In-parallel configuration

When using two oxygenators in parallel, the resistance offered by the oxygenators is reduced since blood flow is shared by two oxygenators. The impact on oxygen content on postoxygenator blood is minimal, as the oxygen content is mainly dependent on hemoglobin oxygen saturation, and this is usually near 100% in all strategies (single oxygenator, association in-series and in-parallel). This phenomenon creates a ceiling effect of oxygen transfer. This finding is consistent with the concept of “rated flow” of the membrane. We used a Quadrox D oxygenator, with a rated flow close to 7L/minute. It is possible that the in-parallel association would provide an increase in oxygen content in two different situations, both using the rated flow concept: using an oxygenator with a small surface area and using higher blood flows than 7L/minute. In this last case, the limiting factor for an increase in blood flow is usually associated with venous drainage cannulation, such as the cannula size, length and site used.

As shown in figure 4, the impact of the parallel configuration on CO_2_ removal is substantial. This occurs due to the higher blood solubility of CO_2_ in comparison to oxygen. Carbon dioxide clearance is a function of the membrane lung surface area and the gradient between the inlet PaCO_2_ (venous PaCO_22_) and the concentration of CO_2_ in the sweep gas. Since both configurations double the surface area-to-blood flow relationship, this improvement in decarboxylation is expected.

Using an in-parallel configuration results in lower flow through each oxygenator. Since higher blood flows can be easily supported by oxygenators, the total flow of the circuit will be limited by the venous drainage cannulation strategy and the patient’s hemodynamic status. When higher extracorporeal circuit blood flows cannot be reached, we must be concerned about lower blood flow through the oxygenators and the increased clogging and clotting risks;^(22)^ furthermore, the need to reach adequate anticoagulation is vital.

### In-series configuration

The circuit configuration with two in-series oxygenator associations results in a higher resistance to blood flow compared with the in-parallel strategy. This may result in a lower total blood flow with the same rotation speed or a higher preoxygenator pressure to provide the same blood flow. The impact on the oxygen content in the postoxygenator blood flow is also minimal for the same reasons discussed above. If hypoxemia is persistent due to the patient’s condition, as in high VO_2_, low venous oxygen saturation (SvO_2_) or high cardiac output states, the association of two in-series oxygenators may result in higher patient saturation, also conditional on the substantially increased blood flow. In this scenario, a single oxygenator offers a high resistance because of its intrinsic characteristics; therefore, this in-series association increases the risk of hemolysis.

The CO_2_ removal in this configuration is also higher when compared to a single oxygenator use. As previously discussed, CO_2_ clearance is a function of the preoxygenator PaCO_2_ and the CO_2_ gradient between the preoxygenator and sweep gas. In this way, the second in-series oxygenator may provide additional CO_2_ removal but with a smaller effect than the in-parallel configuration due to a higher exchange surface but with a lower CO_2_ partial pressure at the inlet of the second oxygenator.

We must highlight that the maintenance of sweep gas flow at the same value when modeling single and two oxygenators (in-parallel or in-series) is of high importance to reach our results. Reducing the sweep flow to half, to keep the same volume of air passing through the oxygenators per time unit when associated, in relation to the blood volume passage, will probably flatten the benefit in reducing the CO_2_, since CO_2_ is 18 times more diffusible than oxygen, and for this reason, its exchange is more dependent on the countercurrent or concurrent air and not on the membrane contact surface.^(4,5)^ Otherwise, from a practical point of view, there is no reason to reduce the sweep flow. In conclusion, the elevation of sweep flow per se can enhance CO_2_ transfer without the placement of a new oxygenator; therefore, the association of a second oxygenator must be reserved for high sweep gas flow refractory clinical significant case of hypercapnia.

### Resistance

Finally, an interesting finding of this study was the nonlinear relationship between blood flow and resistance, with a higher resistance at lower and higher flows and a lower resistance with intermediate blood flows, forming a U shape. This finding could be explained by an inherent resistance of higher blood flows and by the inertia of the blood and a possible closing pressure of the oxygenator fibers at low blood flows. In the former hypothesis, since the blood flow through the oxygenators is increased, fiber separation results in lower resistance.

### Previous literature

There are few case reports previously published using two associated oxygenators in patients with refractory hypoxemia using VV-ECMO.^(14-16)^ Kang et al. described the use of two oxygenators in series in an obese patient with refractory hypoxemia and hypercapnia, which improved after achieving a blood flow of 10.2L/minute through the two oxygenators.^(14)^ Leloup et al. also described an in-series oxygenator association in an ARDS patient evolving with refractory hypercapnia and concomitant traumatic cerebral hemorrhage. This patient needed a blood flow of 5.1L/minute through the oxygenators to resolve the hypercapnia; however, there was very little effect on her systemic oxygenation.^(15)^ Cantwel et al. described a leptospirosis patient with alveolar hemorrhage who needed ECMO support, in which the in-parallel configuration was used due to refractory hypoxemia and hypercapnia. A prompt resolution of the hypercapnia was achieved, with a progressive correction of hypoxemia, using an ECMO blood flow up to 8L/minute.^(16)^ In a particular ECMO support scenario, Malik et al. described the use of two ECMO circuits in parallel (with the need for four cannulas), both for exclusive respiratory assistance (two VV-ECMO circuits). The systemic gases improved with a total blood flow of 9L/minute.^(8)^ Navas-Blanco et al. described the successful association of venoarterial and VV-ECMO in a patient with associated severe cardiovascular and respiratory failure.^(23)^ Hamilton et al.,^(10)^ Gygax et al.^(13)^ and Lonsky et al.^(12)^ described the successful use of an in-parallel association of oxygenators during cardiopulmonary bypass in very obese patients. Kelli et al. experimentally described the in-parallel association of oxygenators as effective in improving oxygenation in situations with a low rate of blood flow.^(11)^ Unfortunately, these previous reports included little information about circuit pressures, circuit resistance, postmembrane oxygen content and CO_2_ removal.

In general, oxygenations slightly improve after any type of association of a second oxygenator, but the data reported do not allow us to infer whether this improvement was due to the presence of the second oxygenator or simply due to an increase in blood flow. Hypercapnia, when present, is highly reduced with any of the tested associations. The effect of low-resistance oxygenator associations on pressures in the extracorporeal circuit is minimal.

### Limitations

This study has several limitations. It was performed with an animal model using a type of oxygenator with excellent performance. The use of different types of oxygenators may result in different findings, as resistance to blood flow, surface area, and rated flow may differ according to the model and fabricants. Despite the different characteristics of the oxygenators on the market, they all have reasonable performance considering these characteristics. The animal models had controlled physiological conditions. Patients with acidosis, anemia, hypoxia, and hyperthermia may have different CO_2_ and oxygen kinetics. Therefore, the behavior of oxygenation and CO_2_ clearance may be slightly different in such clinical scenarios. The period studied was very short, and the physiology of the oxygenator changes considerably over time.^(24)^ We collected data from animals using one oxygenator and derived the data for two oxygenators. Finally, although we used a previously validated mathematical model, it is possible that other unmeasured variables that were not considered in the model will alter the results. A direct comparison between the two configurations must be performed to confirm or refute our data.

## CONCLUSION

The use of oxygenators during venous-venous extracorporeal membrane oxygenation support provides a modest increase in carbon dioxide partial pressure removal with a slight improvement in oxygenation. The effect of oxygenator associations on extracorporeal circuit pressures is minimal, but it depends on the intrinsic oxygenator properties. Understanding the limitations of the available products, the hemodynamic status, and the physiology of the patient facilitates the application of these findings; moreover, the use of associations of oxygenators is limited to specific and extreme rescue scenarios.
