# Supplementary material for: Exploring the association of two oxygenators in parallel or in series during respiratory support using extracorporeal membrane oxygenation
Source: Rev Bras Ter Intensiva. 2022 Oct-Dec;34(4):402–9. doi: 10.5935/0103-507X.20220299-en (PMC9987005; doi:10.5935/0103-507X.20220299-en)
Supplement: Supplementary file 1 [file rbti-34-04-0402-suppl1.pdf]

Exploring the association of two oxygenators in parallel or in series during respiratory support using extracorporeal membrane oxygenation

Investigação da associação de dois oxigenadores em paralelo ou em série durante o suporte respiratório com oxigenação por membrana extracorpórea

Livia Maria Garcia Melro[1], Yuri de Albuquerque Pessoa dos Santos[2], Luis Carlos Maia Cardozo Júnior[2], Bruno Adler Maccagnan Pinheiro Besen[1], Rogério Zigaib[1], Daniel Neves Forte[2], Pedro Vitale Mendes[2], Marcelo Park[2]

Table 1S - Characteristics of animals at the baseline and after multiple organ failure induction

	Baseline	MOF	p value*
Temperature	37.50 [37.20 - 38.10]	37.60 [37.30 - 38.40]	0.917
Hemoglobin	11.10 [10.60 - 11.90]	9.80 [8.30 - 10.00]	0.021
Central venous pressure	7.00 [5.00 - 8.00]	16.00 [10.00 - 19.00]	0.036
Blood flow = 500mL/minute			
Venous line pressure (mmHg)	1.00 [-1.00 - 5.00]	3.00 [-2.00 - 4.00]	0.917
Preoxygenator pressure (mmHg)	30.00 [25.00 - 31.00]	30.00 [23.00 - 35.00]	1.000
Postoxygenator pressure (mmHg)	26.00 [24.00 - 28.00]	25.00 [20.00 - 27.00]	0.834
Rotations per minute	1,135.00 [1,000.00 - 1,160.00]	1,075.00 [1,065.00 - 1,125.00]	0.754
Blood flow = 1,000mL/minute			
Venous line pressure (mmHg)	-9.00 [-13.00 - -2.00]	-5.00 [-14.00 - -5.00]	0.917
Preoxygenator pressure (mmHg)	46.00 [43.00 - 52.00]	58.00 [50.00 - 70.00]	0.347
Postoxygenator pressure (mmHg)	40.00 [40.00 - 50.00]	49.00 [42.00 - 66.00]	0.463
Rotations per minute	1,645.00 [1,500.00 - 1,676.00]	1,565.00 [1,515.00 - 1,695.00]	0.917
Blood flow = 1,500mL/minute			
Venous line pressure (mmHg)	-21.00 [-27.00 - -14.00]	-17.00 [-26.00 - -15.00]	0.754
Preoxygenator pressure (mmHg)	75.00 [67.00 - 77.00]	74.00 [70.00 - 80.00]	0.917
Postoxygenator pressure (mmHg)	69.00 [56.00 - 74.00]	68.00 [62.00 - 72.00]	0.917
Rotations per minute	2,060.00 [1,900.00 - 2,140.00]	1,995.00 [1,901.00 - 2,090.00]	0.917
Blood flow = 2,000mL/minute			
Venous line pressure (mmHg)	-35.00 [-35.00 - -27.00]	-29.00 [-39.00 - -28.00]	1.000
Preoxygenator pressure (mmHg)	102.00 [100.00 - 105.00]	102.00 [94.00 - 104.00]	1.000
Postoxygenator pressure (mmHg)	92.00 [85.00 - 100.00]	94.00 [84.00 - 96.00]	0.834
Rotations per minute	2,435.00 [2,300.00 - 2,600.00]	2,425.00 [2,285.00 - 2,465.00]	0.754
Blood flow = 2,500mL/minute			
Venous line pressure (mmHg)	-48.00 [-60.00 - -41.00]	-42.00 [-51.00 - -41.00]	0.834
Preoxygenator (mmHg)	136.00 [133.00 - 142.00]	132.00 [125.00 - 133.00]	0.530
Postoxygenator pressure (mmHg)	126.00 [122.00 - 129.00]	124.00 [111.00 - 126.00]	0.834
Rotations per minute	2,770.00 [2,700.00 - 2,965.00]	2,760.00 [2,635.00 - 2,855.00]	0.602
Blood flow = 3,000mL/minute			
Venous line pressure (mmHg)	-61.00 [-68.00 - -55.00]	-59.00 [-62.00 - -58.00]	1.000
Preoxygenator pressure (mmHg)	172.00 [160.00 - 184.00]	166.00 [162.00 - 171.00]	0.917
Postoxygenator pressure (mmHg)	159.00 [156.00 - 168.00]	159.00 [145.00 - 159.00]	0.751
Rotations per minute	3,060.00 [3,000.00 - 3,280.00]	3,065.00 [3,010.00 - 3,285.00]	0.754
Blood flow = 3,500mL/minute			
Venous line pressure (mmHg)	-76.00 [-88.00 - -76.00]	-75.00 [-82.00 - -75.00]	0.673
Preoxygenator pressure (mmHg)	213.00 [196.00 - 228.00]	216.00 [211.00 - 224.00]	0.754
Postoxygenator pressure (mmHg)	199.00 [192.00 - 202.00]	196.00 [192.00 - 219.00]	1.000
Rotations per minute	3,400.00 [3,380.00 - 3,590.00]	3,525.00 [3,375.00 - 3,590.00]	1.000

Continue...

...continuation

	Baseline	MOF	p value*
Blood flow = 4,000mL/minute			
Venous line pressure (mmHg)	-96.00 [-101.00 - -92.00]	-100.00 [-102.00 - -93.00]	0.602
Preoxygenator pressure (mmHg)	257.00 [235.00 - 278.00]	267.00 [260.00 - 269.00]	0.602
Postoxygenator pressure (mmHg)	241.00 [232.00 - 245.00]	244.00 [238.00 - 261.00]	0.602
Rotations per minute	3,750.00 [3,700.00 - 3,945.00]	3,855.00 [3,765.00 - 3,975.00]	0.602
Blood flow = 4,500mL/minute			
Venous line pressure (mmHg)	-105.00 [-106.00 - -102.00]	-105.00 [-107.00 - -102.00]	0.916
Preoxygenator pressure (mmHg)	313.00 [274.00 - 338.00]	312.00 [280.00 - 327.00]	0.917
Postoxygenator pressure (mmHg)	288.00 [271.00 - 295.00]	293.00 [278.00 - 293.00]	0.753
Rotations per minute	4,100.00 [3,985.00 - 4,300.00]	4,135.00 [4,025.00 - 4,285.00]	0.917
Blood flow = 5,000mL/minute			
Venous line pressure (mmHg)	-105.00 [-106.00 - -105.00]	-105.00 [-107.00 - -105.00]	1.000
Preoxygenator pressure (mmHg)	373.00 [323.00 - 377.00]	367.00 [313.00 - 387.00]	0.917
Postoxygenator pressure (mmHg)	331.00 [321.00 - 337.00]	347.00 [293.00 - 348.00]	0.602
Rotations per minute	4,500.00 [4,330.00 - 4,655.00]	4,515.00 [4,130.00 - 4,650.00]	0.917
Blood flow = 5,500mL/minute			
Venous line pressure (mmHg)	-105.00 [-107.00 - -105.00]	-105.00 [-107.00 - -105.00]	0.827
Preoxygenator pressure (mmHg)	416.00 [378.00 - 420.00]	408.00 [367.00 - 447.00]	0.675
Postoxygenator pressure (mmHg)	374.00 [372.00 - 378.00]	403.00 [344.00 - 403.00]	0.917
Rotations per minute	4,800.00 [4,685.00 - 5,010.00]	4,890.00 [4,335.00 - 5,000.00]	0.754

MOF - multiple organ failure. *Wilcoxon's test between baseline and multiple organ failure. Data are shown as median [percentile 25th - percentile 75th].

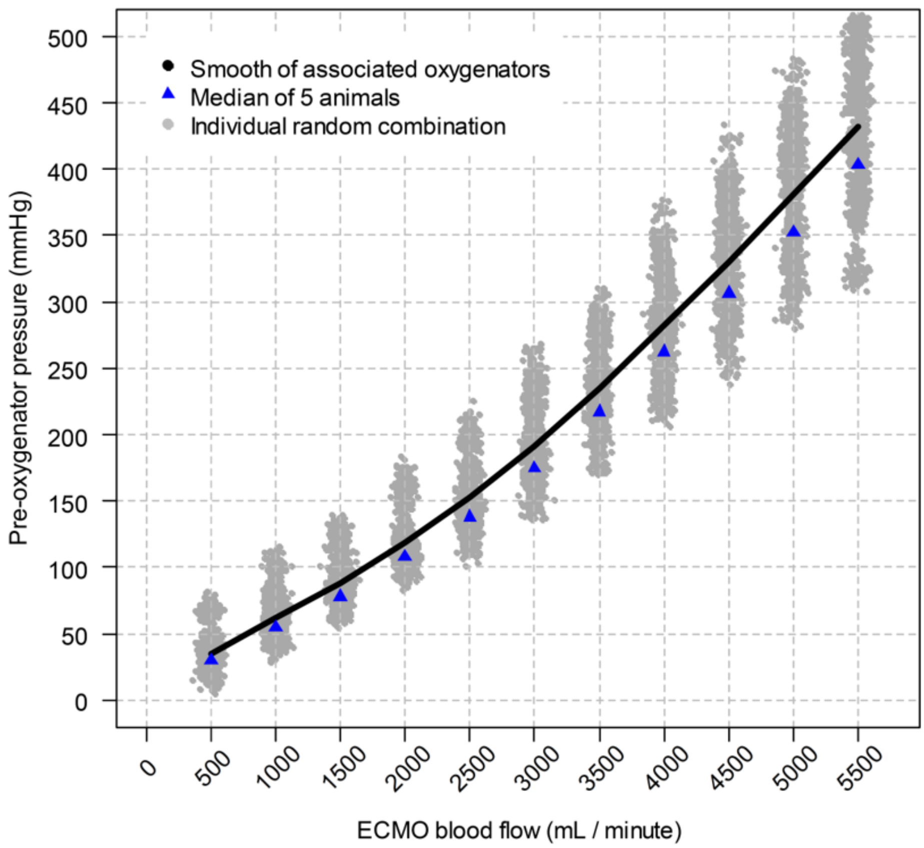

Figure 1S - Median preoxygenator pressure for single oxygenators of five animals and preoxygenator pressures for one hundred random in-series associations of two oxygenators.
For each extracorporeal membrane oxygenation blood flow point, there are one hundred random combinations. ECMO - extracorporeal membrane oxygenation.

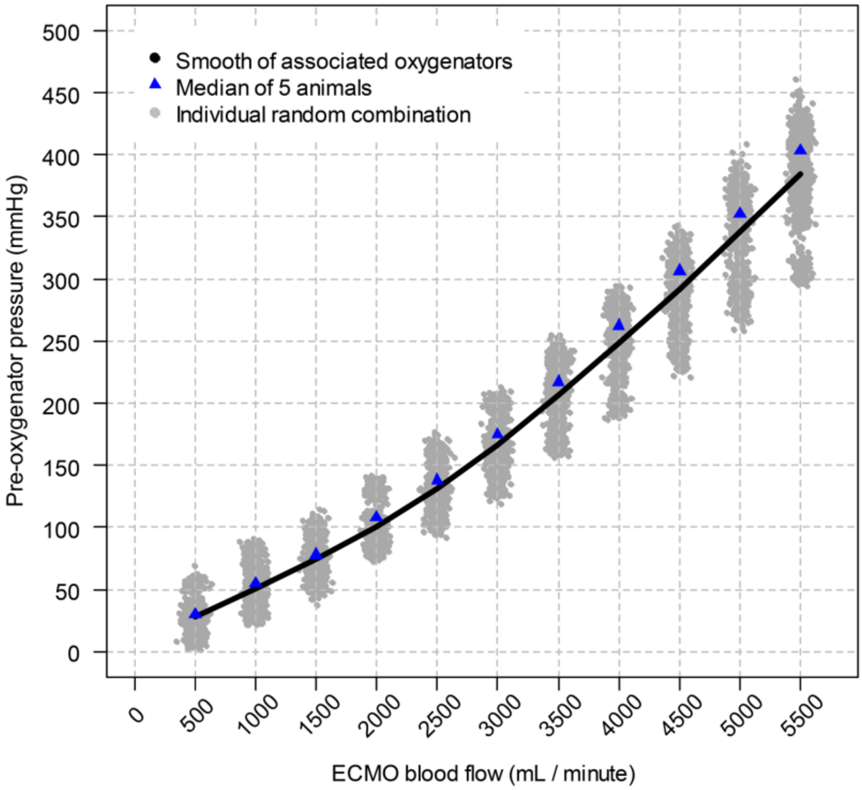

Figure 2S - Median preoxygenator pressure for single oxygenators of five animals and preoxygenator pressures for one hundred random parallel associations of two oxygenators. For each extracorporeal membrane oxygenation blood flow point, there are one hundred random combinations. ECMO - extracorporeal membrane oxygenation.

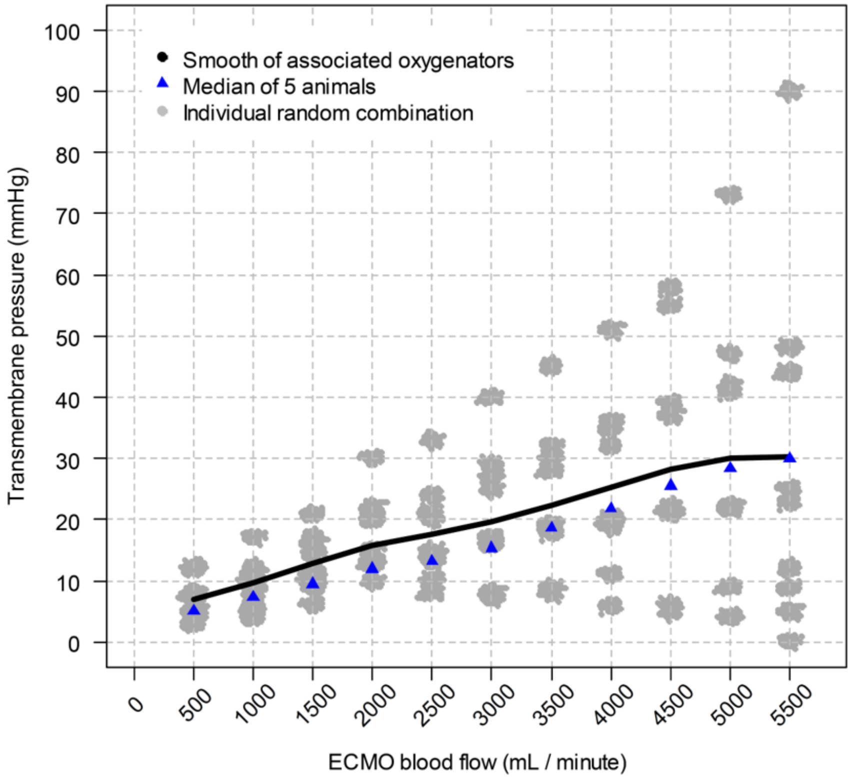

Figure 3S - Median transmembrane pressure for single oxygenators of five animals and transmembrane pressures for one hundred random in-series associations of two oxygenators. For each extracorporeal membrane oxygenation blood flow point, there are one hundred random combinations. ECMO - extracorporeal membrane oxygenation.

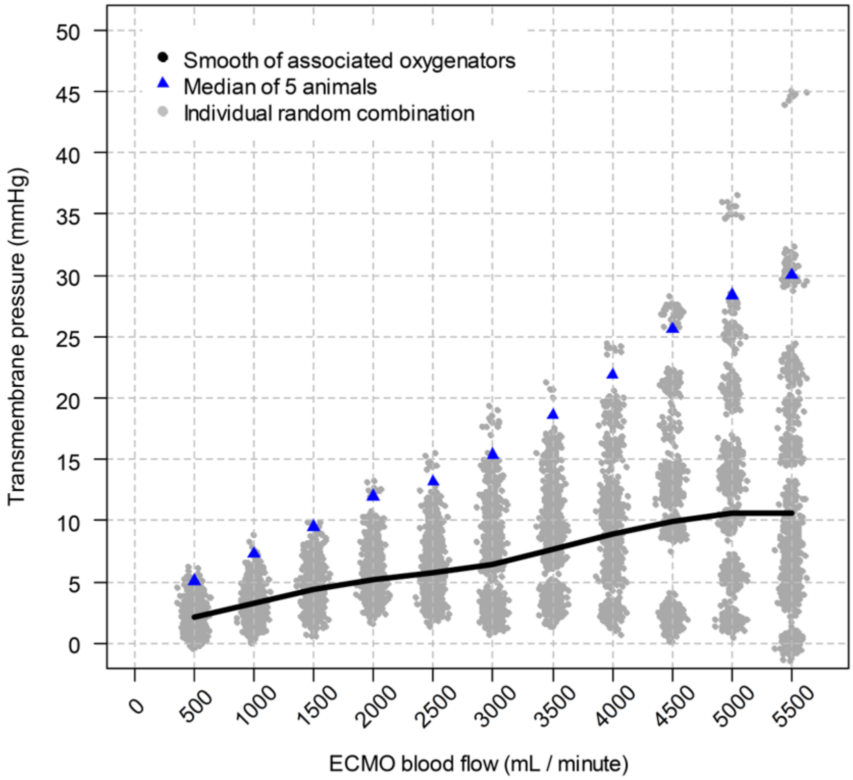

Figure 4S - Median transmembrane pressure for single oxygenators of five animals and transmembrane pressures for one hundred random parallel associations of two oxygenators.

For each extracorporeal membrane oxygenation blood flow point, there are one hundred random combinations. ECMO - extracorporeal membrane oxygenation.

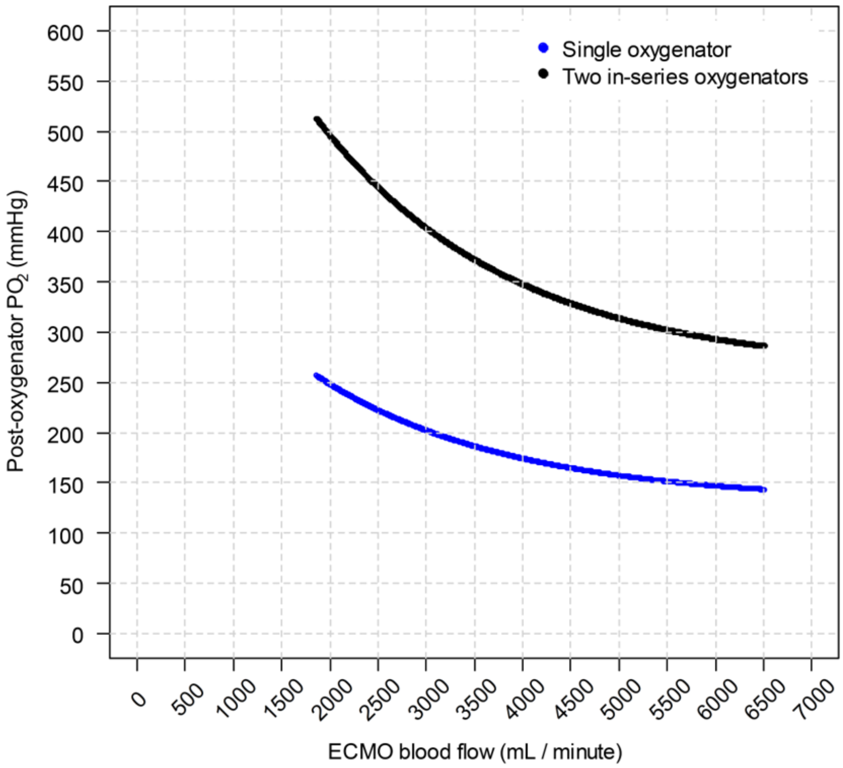

Figure 5S - Postmembrane oxygen partial pressure using a single oxygenator and two in-series oxygenators.

Clinical status of modeling: oxygen consumption of 200mL/minute; hemoglobin level of 10g/dL; cardiac output of 10L/minute; partial pressure of carbon dioxide of 40mmHg; ventilator fraction of inspired oxygen of 30%; pulmonary shunt fraction of 100%; sweep gas flow of 4L/minute; sweep gas fraction of inspired oxygen of 100%. PO_2 - partial pressure of oxygen; ECMO - extracorporeal membrane oxygenation.

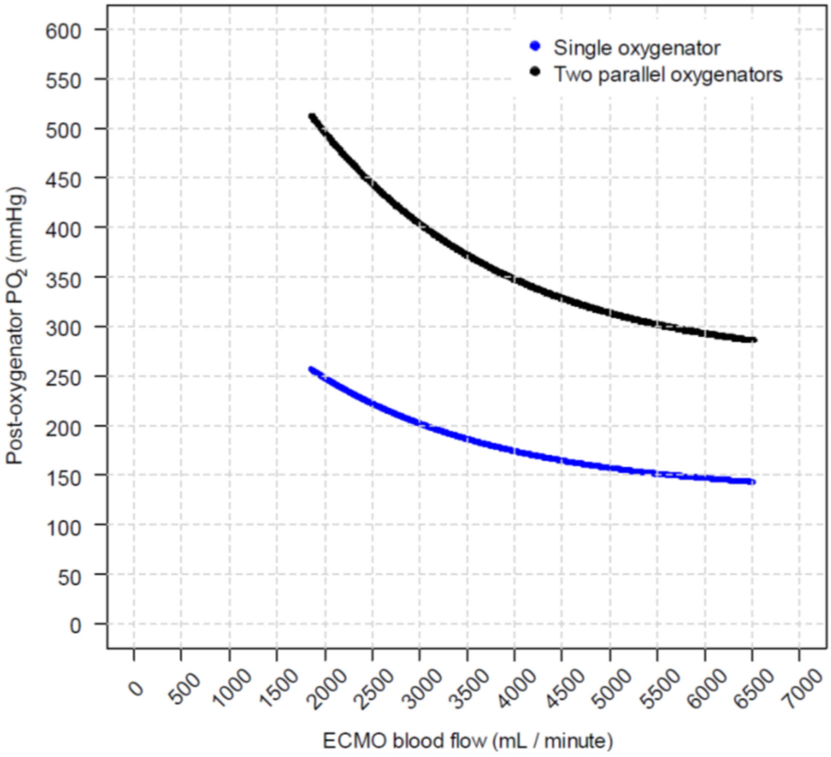

Figure 6S - Postoxygenator oxygen partial pressure using a single oxygenator and two parallel oxygenators.

Clinical status of modeling: oxygen consumption of 200mL/minute; hemoglobin level of 10 g/dL; cardiac output of 10L/minute; partial pressure of carbon dioxide of 40mmHg; ventilator fraction of inspired oxygen of 30%; pulmonary shunt fraction of 100%; sweep gas flow of 4L/minute; sweep gas fraction of inspired oxygen of 100%. PO_2 - oxygen partial pressure; ECMO - extracorporeal membrane oxygenation.

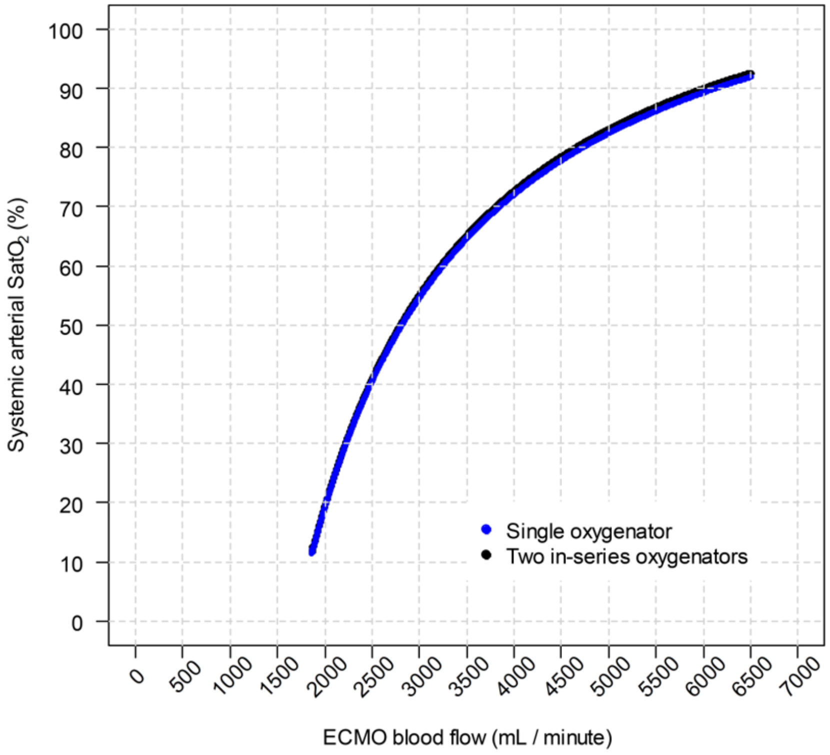

Figure 7S - Systemic arterial oxygen saturation using a single oxygenator and two in-series oxygenators in higher oxygen consumption setting.

Clinical status of modeling: oxygen consumption of 300mL/minute; hemoglobin level of 10g/dL; cardiac output of 10L/minute; partial pressure of carbon dioxide of 40mmHg; ventilator fraction of inspired oxygen of 30%; pulmonary shunt fraction of 100%; sweep gas flow of 4L/minute; sweep gas fraction of inspired oxygen of 100%. $SatO_2$ - arterial oxygen saturation; ECMO - extracorporeal membrane oxygenation.

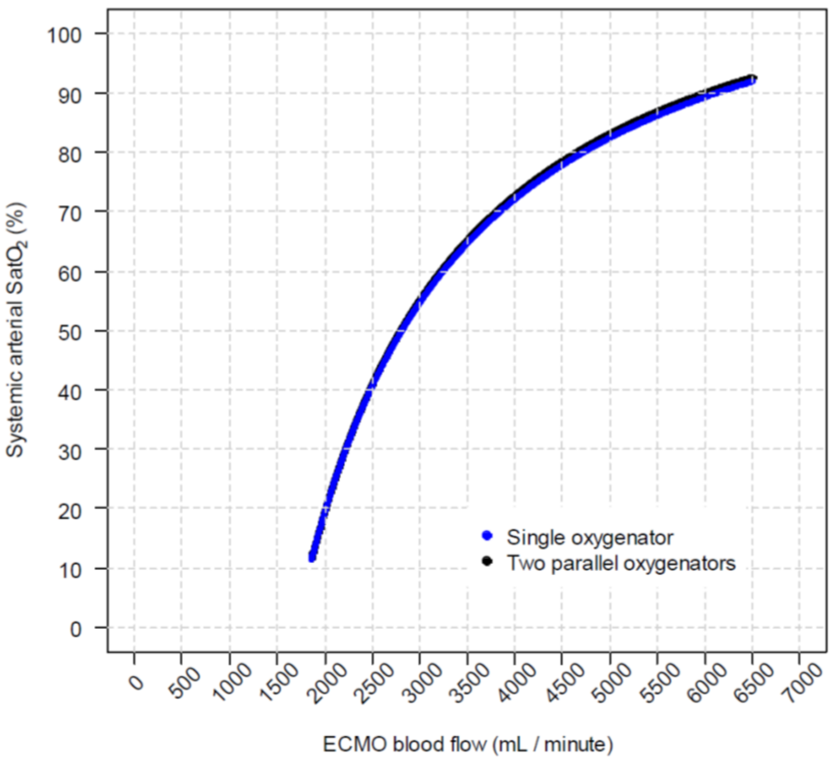

Figure 8S - Systemic arterial oxygen saturation using a single oxygenator and two parallel oxygenators in higher oxygen consumption setting.

Clinical status of modeling: oxygen consumption of 300mL/minute; hemoglobin level of 10g/d; cardiac output of 10L/minute; partial pressure of carbon dioxide of 40mmHg; ventilator fraction of inspired oxygen of 30%; pulmonary shunt fraction of 100%; sweep gas flow of 4 L/minute; sweep gas fraction of inspired oxygen of 100%. $SatO_2$ - arterial oxygen saturation; ECMO - extracorporeal membrane oxygenation.
